# Wettability of semispherical droplets on layered elastic gradient soft substrates

**DOI:** 10.1038/s41598-020-80575-9

**Published:** 2021-01-26

**Authors:** Yonglin Yang, Xing Li, Wenshuai Wang

**Affiliations:** School of Mathematics and Statistics, Ningxia University, Yinchuan, 750021 China

**Keywords:** Materials science, Physics

## Abstract

Research on the wettability of soft matter is one of the most urgently needed studies in the frontier domains, of which the wetting phenomenon of droplets on soft substrates is a hot subject. Scholars have done considerable studies on the wetting phenomenon of single-layer structure, but it is noted that the wetting phenomenon of stratified structure is ubiquitous in nature, such as oil exploitation from geological structural layers and shale gas recovery from shale formations. Therefore, the wettability of droplets on layered elastic gradient soft substrate is studied in this paper. Firstly, considering capillary force, elastic force and surface tension, the constitutive equation of the substrate in the vector function system is derived by using the vector function system in cylindrical coordinates, and the transfer relation of layered structure is obtained. Further, the integral expressions of displacement and stress of double Bessel function are given. Secondly, the numerical results of displacement and stress are obtained by using the numerical formula of double Bessel function integral. The results show that the deformation of the substrate weakens with the increase of the elastic modulus, also the displacement and stress change dramatically near the contact line, while the variation is flat when the contact radius is far away from the droplet radius.

## Introduction

In 1991, P.G. de Gennes, a famous French physicist, gave a lecture on "soft matter" at the Nobel Prize-giving Conference, and the word "soft matter" was first explicitly put forward^1^. Soft matter, including liquid crystal, polymer, colloid, membrane, foam, droplet, granular matter, life system and so on, can be the solid–liquid phase mixtures, liquid–liquid mixtures or liquid–gas mixtures, etc.^2,3^, which are widely found in nature, daily life and production activities. The distinguished difference between hard matter and soft matter is that the former produces smaller deformation under large stress, while the latter produces large deformation under small stress. Thus, the obvious characteristic of soft matter is very sensitive to external stimuli. More importantly, the problems of surface and interface of soft matter have become one of the key areas of interdisciplinary research in physics, chemistry, biology and mathematics, especially the phenomenon of surface wetting.

Thomas Young, the pioneer of wetting science research, come up with the idea of contact angle in 1805, so as to characterize the surface wetting stability^4^. From then on, scholars have carried out a lot of researches on wetting phenomenon^5–14^. It can be seen that this phenomenon is ubiquitous and significantly important, such as oil exploitation, printing and painting^5,7^, application of superhydrophobic substrates^8–10^ and so on. On an enough soft substrate, static droplets will cause significant deformation of the substrate, which is driven by capillary force on the contact line and fluid pressure on the solid surface. Meanwhile, on the surface, these forces are balanced via solid traction stress caused by the substrate deformation^15^. Surface tension usually makes no negligible difference in large scale, but it likely plays a significant role on micro-nano scale or very flexible objects^16^. In many natural phenomena, capillary force of liquid, elastic deformation of solid structure and surface tension are highly coupled. Based on these theories, many scholars have studied the coupling effect of some basic microstructures and capillary force. For example, Liu et al.^17^ used the basic solution of three-dimensional elasticity to analyze the deformation field of soft substrate under the action of liquid droplets. Gurtin et al.^18^ put forward the theory of surface elasticity, from which the influence of surface residual stress and surface elasticity on the structure can be expressed by Young–Laplace equation and surface layer model^19^. Therefore, it is necessary to consider the influences of capillary force, elastic force and surface tension on the substrate.

There were many researchers devoting on the wetting phenomenon of single-layer isotropic materials in model, experiment and theory^15,20–30^, but the wetting phenomena of layered structure were rarely studied. In fact, many natural phenomena are related to the wetting of the layered structure. For example, micro- and nano-layered structure has been proved to play an important role in obtaining high apparent contact angle and low adhesion of lotus leaves^31^, and the geological structure of oil exploitation is also layered. The water and ice repellent properties of layered flexible superhydrophobic substrate composed of poly (dimethylsiloxane) and zinc oxide were studied, and this study offered insights into the design of multi-structured materials^32^. So it had potential application value to study the wettability of layered structure. It is noted that most anisotropic materials can be synthesized by lamination technology, so layered isotropy can be used to approximate the wetting condition of anisotropy. For layered elastic structures, Pan et al.^33,34^ expressed the displacement and stress by vector function system, and gave the transfer matrix of layered elastic structure by using the transfer matrix method, then solved the displacement and stress of each layer. In this paper, we follow Pan’s work and develop their method into the wetting phenomenon, and study the deformation of elastic layered isotropic thin substrate caused by hemispherical droplets without considering the gravity of droplet under micro-scale. The partial differential equation can be transformed into ordinary differential matrix equation by algebraic operation when the displacement and stress are expressed by vector function system, and then the inter-layer displacement and stress are given by using transfer relation. Finally, the expressions of displacement and stress at any field point are given by using layered technology. The results have potential application value in designing and fabricating soft devices.

## Model establishment and basic equations

**Figure 1:**
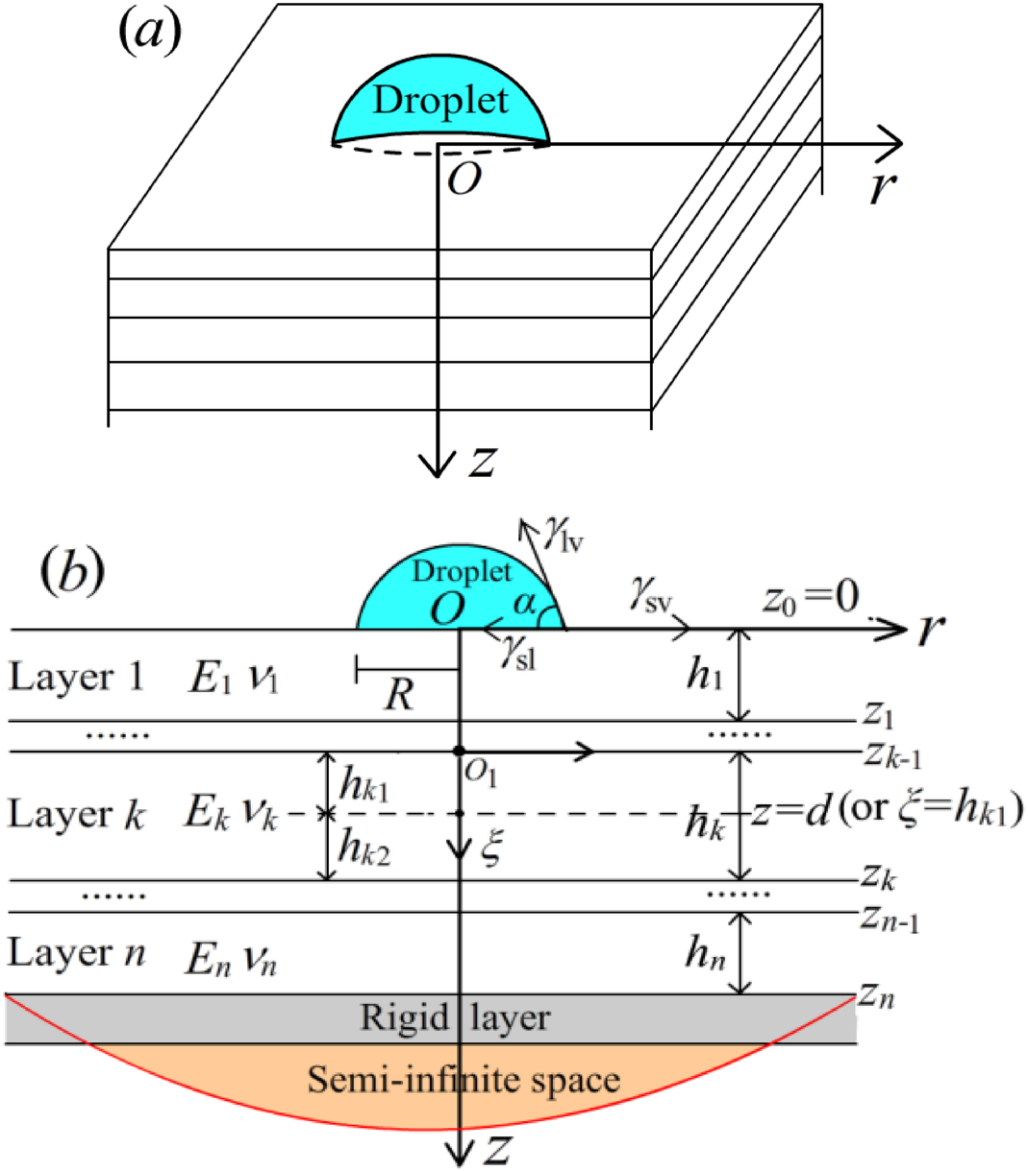
The sketch of droplet wetting with layered structure.

The semispherical droplet is placed on a layered elastic soft substrate, and the bottom is fixed with a rigid surface or a semi-infinite space. It is assumed that the layered structure is not affected by body force, and the layers are in complete contact, that is the displacement and stress at the interface are continuous, and the thickness, elastic modulus and Poisson's ratio of each layer are known. The contact model of elastic substrate deformation caused by equilibrium droplet is established, where the deformation of substrate is axisymmetric, as shown in Fig. 1.

In the cylindrical coordinate system $\left( {r,\theta ,z} \right)$, the equilibrium equation of layered elastic medium is

1 $$\begin{gathered} \frac{{\partial \sigma_{rr} }}{\partial r} + \frac{{\partial \sigma_{r\theta } }}{r\partial \theta } + \frac{{\partial \sigma_{rz} }}{\partial z} + \frac{{\sigma_{rr} - \sigma_{\theta \theta } }}{r} = 0, \hfill \\ \frac{{\partial \sigma_{r\theta } }}{\partial r} + \frac{{\partial \sigma_{\theta \theta } }}{r\partial \theta } + \frac{{\partial \sigma_{\theta z} }}{\partial z} + \frac{{2\sigma_{r\theta } }}{r} = 0, \hfill \\ \frac{{\partial \sigma_{rz} }}{\partial r} + \frac{{\partial \sigma_{\theta z} }}{r\partial \theta } + \frac{{\partial \sigma_{zz} }}{\partial z} + \frac{{2\sigma_{rz} }}{r} = 0, \hfill \\ \end{gathered}$$

where $\sigma_{ij}$ is the stress components. The geometric equation is

2 $$\begin{gathered} \varepsilon_{rr} = \frac{{\partial u_{r} }}{\partial r},\varepsilon_{\theta \theta } = \frac{{\partial u_{\theta } }}{r\partial \theta } + \frac{{u_{r} }}{r},\varepsilon_{zz} = \frac{{\partial u_{z} }}{\partial z}, \hfill \\ \varepsilon_{\theta z} = \frac{1}{2}\left( {\frac{{\partial u_{\theta } }}{\partial z} + \frac{{\partial u_{z} }}{r\partial \theta }} \right), \hfill \\ \varepsilon_{rz} = \frac{1}{2}\left( {\frac{{\partial u_{z} }}{\partial r} + \frac{{\partial u_{r} }}{\partial z}} \right), \hfill \\ \varepsilon_{r\theta } = \frac{1}{2}\left( {\frac{{\partial u_{r} }}{r\partial \theta } + \frac{{\partial u_{\theta } }}{\partial r} - \frac{{u_{\theta } }}{r}} \right), \hfill \\ \end{gathered}$$

where $\varepsilon_{ij} ,u_{i}$ are strain components and displacement components, respectively. The constitutive relation of layered isotropic materials in cylindrical coordinate system is

3 $$\begin{gathered} \sigma_{rr} = c_{1} \varepsilon_{rr} + c_{2} \varepsilon_{\theta \theta } + c_{2} \varepsilon_{zz} , \hfill \\ \sigma_{\theta \theta } = c_{2} \varepsilon_{rr} + c_{1} \varepsilon_{\theta \theta } + c_{2} \varepsilon_{zz} , \hfill \\ \sigma_{zz} = c_{2} \varepsilon_{rr} + c_{2} \varepsilon_{\theta \theta } + c_{1} \varepsilon_{zz} , \hfill \\ \sigma_{ij} = 2c_{3} \varepsilon_{ij} ,\;\left( {i \ne j\;;\;i,j = r,\theta ,z} \right), \hfill \\ \end{gathered}$$

with

4 $$c_{1} = \frac{{E\left( {1 - \nu } \right)}}{{\left( {1 + \nu } \right)\left( {1 - 2\nu } \right)}},c_{2} = \frac{E\nu }{{\left( {1 + \nu } \right)\left( {1 - 2\nu } \right)}},c_{3} = \frac{E}{{2\left( {1 + \nu } \right)}},\;\;(\nu \ne 0.5),$$

where *E* is the elastic modulus, *ν* is the Poisson's ratio, and the following relation is given by

5 $$c_{1} - c_{2} = 2c_{3} .$$

The inter-layer contact conditions are as follows

$$u_{i}^{ - } \left( {r,\theta ,h_{k} } \right) = u_{i}^{ + } \left( {r,\theta ,h_{k} } \right),\;\sigma_{iz}^{ - } \left( {r,\theta ,h_{k} } \right) = \sigma_{iz}^{ + } \left( {r,\theta ,h_{k} } \right),\;\;\left( {k = 1,2, \cdot \cdot \cdot ,n} \right),$$

where $u_{i}^{ - } \left( {r,\theta ,h_{k} } \right),u_{i}^{ + } \left( {r,\theta ,h_{k} } \right)$ denote the displacements of the upper surface of the *k*-layer and the lower surface of the (*k − *1)-layer, respectively, and the stress has similar expression.

In this paper, the deformation of the substrate caused by only the surface normal load is considered, and the stress boundary conditions can be expressed as

6 $$\sigma_{rz} \left( {r,\theta ,0} \right) = \sigma_{\theta z} \left( {r,\theta ,0} \right) = 0,\;\;\sigma_{zz} \left( {r,\theta ,0} \right) = - \gamma_{lv} \delta \left( {r - R} \right) + PH\left( {R - r} \right),$$

where $\delta \left( x \right)$ and $H\left( x \right)$ are Dirac delta function and Heaviside step function, respectively, *P* is the Laplace pressure inside the droplet. For semispherical droplets,$P = {{2\gamma_{lv} } \mathord{\left/ {\vphantom {{2\gamma_{lv} } R}} \right. \kern-\nulldelimiterspace} R}$, where *R* is the droplet radius, and $\gamma_{lv}$ is the liquid–air surface tension. Since the bottom surface is fixed on a rigid surface or half space, the displacement boundary conditions are

$$u_{i} \left( {r,\theta ,z_{n} } \right) = 0,\;\left( {i = r,\theta ,z} \right).$$

## Constitutive equation under vector function system

The vector functions in cylindrical coordinate system can be expressed as^33,34^

7 $$\begin{gathered} {\varvec{L}}\left( {r,\theta ;\lambda ,m} \right) = {\varvec{e}}_{z} S\left( {r,\theta ;\lambda ,m} \right), \hfill \\ {\varvec{M}}\left( {r,\theta ;\lambda ,m} \right) = \left( {{\varvec{e}}_{r} \frac{\partial }{\partial r} + {\varvec{e}}_{\theta } \frac{\partial }{r\partial \theta }} \right)S\left( {r,\theta ;\lambda ,m} \right), \hfill \\ {\varvec{N}}\left( {r,\theta ;\lambda ,m} \right) = \left( {{\varvec{e}}_{r} \frac{\partial }{r\partial \theta } - {\varvec{e}}_{\theta } \frac{\partial }{\partial r}} \right)S\left( {r,\theta ;\lambda ,m} \right), \hfill \\ \end{gathered}$$

where ${\varvec{e}}_{r} ,{\varvec{e}}_{\theta } ,{\varvec{e}}_{z}$ are unit vectors in $r,\theta ,z$ directions, and *S* satisfies Helmholtz function, i.e.

8 $$S\left( {r,\theta ;\lambda ,m} \right) = \frac{1}{{\sqrt {2\pi } }}J_{m} \left( {\lambda r} \right)e^{jm\theta } ;\;\;m = 0, \pm 1, \pm 2 \ldots ,$$

and

9 $$\frac{{\partial^{2} S}}{{\partial r^{2} }} + \frac{\partial S}{{r\partial r}} + \frac{{\partial^{2} S}}{{r^{2} \partial \theta^{2} }} + \lambda^{2} S = 0,$$

where $J_{m} \left( {\lambda r} \right)$ is the *m*-th order Bessel function of the first kind, $j = \sqrt { - 1}$, and $\lambda ,m$ correspond to the transformation variables of horizontal physical variables $r,\theta$. $m = 0$ corresponds to axisymmetric deformation. For brevity, let

10 $$S\left( {r,\theta ;\lambda ,m} \right) = \frac{1}{{\sqrt {2\pi } }}J_{0} \left( {\lambda r} \right) \triangleq S\left( {r,\theta } \right),$$

11 $$\begin{aligned} & {\varvec{L}}\left( {r,\theta ;\lambda ,m} \right) = {\varvec{e}}_{z} S\left( {r,\theta } \right) \triangleq {\varvec{L}}\left( {r,\theta } \right), \\ & {\varvec{M}}\left( {r,\theta ;\lambda ,m} \right) = \left( {{\varvec{e}}_{r} \frac{\partial }{\partial r} + {\varvec{e}}_{\theta } \frac{\partial }{r\partial \theta }} \right)S\left( {r,\theta } \right) \triangleq {\varvec{M}}\left( {r,\theta } \right), \\ & {\varvec{N}}\left( {r,\theta ;\lambda ,m} \right) = \left( {{\varvec{e}}_{r} \frac{\partial }{r\partial \theta } - {\varvec{e}}_{\theta } \frac{\partial }{\partial r}} \right)S\left( {r,\theta } \right) \triangleq {\varvec{N}}\left( {r,\theta } \right). \\ \end{aligned}$$

Due to the orthogonality and completeness of the vector function system, the displacement and traction vectors of any field point on every layer can be expanded as follows^33^:

12 $$\begin{aligned} \user2{u}\left( {r,\theta ,z} \right) & {\text = }u_{r} \user2{e}_{r} + u_{\theta } \user2{e}_{\theta } + u_{z} \user2{e}_{z} \\ & = \sum\limits_{m} {\int_{0}^{{ + \infty }} {\left[ {U_{L} (z)\user2{L}(r,\theta ) + U_{M} (z)\user2{M}(r,\theta ) + U_{N} (z)\user2{N}(r,\theta )} \right]} } \lambda d\lambda , \\ \end{aligned}$$

13 $$\begin{aligned} \user2{t}\left( {r,\theta ,z} \right) & = \sigma _{{rz}} \user2{e}_{r} + \sigma _{{\theta z}} \user2{e}_{\theta } + \sigma _{{zz}} \user2{e}_{z} \\ & = \sum\limits_{m} {\int_{0}^{{ + \infty }} {\left[ {T_{L} \left( z \right)\user2{L}\left( {r,\theta } \right) + T_{M} \left( z \right)\user2{M}\left( {r,\theta } \right) + T_{N} \left( z \right)\user2{N}\left( {r,\theta } \right)} \right]} } \lambda d\lambda , \\ \end{aligned}$$

where $U_{K} ,T_{K} \;(K = L,M,N)$ are the expansion coefficients of displacement and stress vector functions in cylindrical coordinates, respectively. These values can be expressed as^35^

14 $$\begin{aligned} & U_{L} \left( {\lambda ,z} \right){\text = }\int\limits_{0}^{{2\pi }} {\int\limits_{0}^{\infty } {\user2{u}(r,\theta ,z) \cdot } } \user2{\bar{L}}(r,\theta )rdrd\theta , \\ & U_{M} \left( {\lambda ,z} \right){\text = }\frac{1}{{\lambda ^{2} }}\int\limits_{0}^{{2\pi }} {\int\limits_{0}^{\infty } {\user2{u}(r,\theta ,z) \cdot } } \user2{\bar{M}}(r,\theta )rdrd\theta , \\ & U_{N} \left( {\lambda ,z} \right){\text = }\frac{1}{{\lambda ^{2} }}\int\limits_{0}^{{2\pi }} {\int\limits_{0}^{\infty } {\user2{u}(r,\theta ,z) \cdot } } \user2{\bar{N}}(r,\theta )rdrd\theta , \\ \end{aligned}$$

15 $$\begin{aligned} & T_{L} \left( {\lambda ,z} \right){\text = }\int\limits_{0}^{{2\pi }} {\int\limits_{0}^{\infty } {\user2{t}(r,\theta ,z) \cdot } } \user2{\bar{L}}(r,\theta )rdrd\theta , \\ & T_{M} \left( {\lambda ,z} \right){\text = }\frac{1}{{\lambda ^{2} }}\int\limits_{0}^{{2\pi }} {\int\limits_{0}^{\infty } {\user2{t}(r,\theta ,z) \cdot } } \user2{\bar{M}}(r,\theta )rdrd\theta , \\ & T_{N} \left( {\lambda ,z} \right){\text = }\frac{1}{{\lambda ^{2} }}\int\limits_{0}^{{2\pi }} {\int\limits_{0}^{\infty } {\user2{t}(r,\theta ,z) \cdot } } \user2{\bar{N}}(r,\theta )rdrd\theta , \\ \end{aligned}$$

where the horizontal line above vectors represents the complex conjugate vector. From Eqs. (7), (12) and (13), the expressions of the transformation domain of displacement and stress are as follows

16 $$\begin{aligned} & u_{r} = \sum\limits_{m} {\int_{0}^{ + \infty } {\left[ {U_{M} (z)\frac{\partial S}{{\partial r}} + U_{N} (z)\frac{\partial S}{{r\partial \theta }}} \right]} } \lambda d\lambda , \\ & u_{\theta } = \sum\limits_{m} {\int_{0}^{ + \infty } {\left[ {U_{M} (z)\frac{\partial S}{{r\partial \theta }} - U_{N} (z)\frac{\partial S}{{\partial r}}} \right]} } \lambda d\lambda , \\ & u_{z} = \sum\limits_{m} {\int_{0}^{ + \infty } {U_{L} (z)S} } \lambda d\lambda , \\ \end{aligned}$$

17 $$\begin{aligned} & \sigma _{{rz}} {\text = }\sum\limits_{m} {\int_{0}^{{ + \infty }} {\left[ {T_{M} (z)\frac{{\partial S}}{{\partial r}} + T_{N} (z)\frac{{\partial S}}{{r\partial \theta }}} \right]} } \lambda d\lambda , \\ & \sigma _{{\theta z}} {\text = }\sum\limits_{m} {\int_{0}^{{ + \infty }} {\left[ {T_{M} (z)\frac{{\partial S}}{{r\partial \theta }} - T_{N} (z)\frac{{\partial S}}{{\partial r}}} \right]} } \lambda d\lambda , \\ & \sigma _{{zz}} {\text = }\sum\limits_{m} {\int_{0}^{{ + \infty }} {T_{L} (z)S} } \lambda d\lambda . \\ \end{aligned}$$

From formula (16), we can get

18 $$\begin{aligned} \frac{{\partial u_{r} }}{\partial r} & = \frac{\partial }{\partial r}\left( {\sum\limits_{m} {\int_{0}^{ + \infty } {\left[ {U_{M} (z)\frac{\partial S}{{\partial r}} + U_{N} (z)\frac{\partial S}{{r\partial \theta }}} \right]} } \lambda d\lambda } \right) \\ & = \sum\limits_{m} {\int_{0}^{ + \infty } {\left[ {U_{M} (z)\frac{{\partial^{2} S}}{{\partial r^{2} }} + U_{N} (z)\frac{{\partial^{2} S}}{r\partial r\partial \theta } - U_{N} (z)\frac{\partial S}{{r^{2} \partial \theta }}} \right]} } \lambda d\lambda \\ \frac{{\partial u_{\theta } }}{\partial \theta } & = \frac{\partial }{\partial \theta }\left( {\sum\limits_{m} {\int_{0}^{ + \infty } {\left[ {U_{M} (z)\frac{\partial S}{{r\partial \theta }} - U_{N} (z)\frac{\partial S}{{\partial r}}} \right]} } \lambda d\lambda } \right) \\ & = \sum\limits_{m} {\int_{0}^{ + \infty } {\left[ {U_{M} (z)\frac{{\partial^{2} S}}{{r\partial \theta^{2} }} - U_{N} (z)\frac{{\partial^{2} S}}{\partial \theta \partial r}} \right]} } \lambda d\lambda \\ \frac{{\partial u_{z} }}{\partial z} & = \frac{\partial }{\partial z}\left( {\sum\limits_{m} {\int_{0}^{ + \infty } {U_{L} (z)S} } \lambda d\lambda } \right) = \sum\limits_{m} {\int_{0}^{ + \infty } {\frac{{dU_{L} (z)}}{dz}S} } \lambda d\lambda \\ \end{aligned}$$

Considering gradient materials with exponential modulus, the material constants are expressed as $$c_{1} e^{az} ,$$$$c_{2} e^{az}$$ and $$c_{3} e^{az}$$. By substituting the expressions of geometric equation and Eq. (16) into the constitutive equation, the constitutive relations under vector function system are obtained by

19 $$\begin{aligned} \sigma_{rr} & = c_{1} e^{az} \sum\limits_{m} {\int_{0}^{ + \infty } {\left[ {U_{M} (z)\frac{{\partial^{2} S}}{{\partial r^{2} }} + U_{N} (z)\frac{{\partial^{2} S}}{r\partial r\partial \theta } - U_{N} (z)\frac{\partial S}{{r^{2} \partial \theta }}} \right]} } \lambda d\lambda \\ & \quad + c_{2} e^{az} \sum\limits_{m} {\int_{0}^{ + \infty } {\left[ {U_{M} (z)\frac{{\partial^{2} S}}{{r^{{2}} \partial \theta^{2} }} - U_{N} (z)\frac{{\partial^{2} S}}{r\partial \theta \partial r}{ + }U_{M} (z)\frac{\partial S}{{r\partial r}} + U_{N} (z)\frac{\partial S}{{r^{2} \partial \theta }}} \right]} } \lambda d\lambda \\ & \quad + c_{{2}} e^{az} \sum\limits_{m} {\int_{0}^{ + \infty } {\frac{{dU_{L} (z)}}{dz}S} } \lambda d\lambda , \\ \end{aligned}$$

20 $$\begin{aligned} \sigma_{\theta \theta } & = c_{2} e^{az} \sum\limits_{m} {\int_{0}^{ + \infty } {\left[ {U_{M} (z)\frac{{\partial^{2} S}}{{\partial r^{2} }} + U_{N} (z)\left( {\frac{{\partial^{2} S}}{r\partial r\partial \theta } - \frac{\partial S}{{r^{2} \partial \theta }}} \right)} \right]} } \lambda d\lambda \\ & \quad + c_{1} e^{az} \sum\limits_{m} {\int_{0}^{ + \infty } {\left[ {U_{M} (z)\left( {\frac{{\partial^{2} S}}{{r^{2} \partial \theta^{2} }} + \frac{\partial S}{{r\partial r}}} \right) - U_{N} (z)\left( {\frac{{\partial^{2} S}}{r\partial \theta \partial r} - \frac{\partial S}{{r^{2} \partial \theta }}} \right)} \right]} } \lambda d\lambda \\ & \quad + c_{{2}} e^{az} \sum\limits_{m} {\int_{0}^{ + \infty } {\frac{{dU_{L} (z)}}{dz}S} } \lambda d\lambda , \\ \end{aligned}$$

21 $$\sigma_{zz} = e^{az} \sum\limits_{m} {\int_{0}^{ + \infty } {\left[ { - c_{{2}} U_{M} (z)\lambda^{2} S + c_{{1}} \frac{{dU_{L} (z)}}{dz}S} \right]} } \lambda d\lambda ,$$

22 $$\sigma_{\theta z} = c_{{3}} e^{az} \sum\limits_{m} {\int_{0}^{ + \infty } {\left[ {\frac{{dU_{M} (z)}}{dz}\frac{\partial S}{{r\partial \theta }} - \frac{{dU_{N} (z)}}{dz}\frac{\partial S}{{\partial r}} + U_{L} (z)\frac{\partial S}{{r\partial \theta }}} \right]} } \lambda d\lambda ,$$

23 $$\sigma_{rz} = c_{{3}} e^{az} \sum\limits_{m} {\int_{0}^{ + \infty } {\left[ {\frac{{dU_{M} (z)}}{dz}\frac{\partial S}{{\partial r}} + \frac{{dU_{N} (z)}}{dz}\frac{\partial S}{{r\partial \theta }} + U_{L} (z)\frac{\partial S}{{\partial r}}} \right]} } \lambda d\lambda ,$$

24 $$\sigma_{r\theta } = c_{{3}} e^{az} \sum\limits_{m} {\int_{0}^{ + \infty } {\left[ {2U_{M} (z)\left( {\frac{{\partial^{2} S}}{r\partial \theta \partial r} - \frac{\partial S}{{r^{2} \partial \theta }}} \right) + U_{N} (z)\left( {\frac{{\partial^{2} S}}{{r^{2} \partial \theta^{2} }} - \frac{{\partial^{2} S}}{{\partial r^{2} }} + \frac{\partial S}{{r\partial r}}} \right)} \right]} } \lambda d\lambda .$$

Comparing Eqs. (21)–(23) with Eq. (17) yields

25 $$\left\{ {\begin{array}{*{20}c} {\left[ {T_{M} (z) - c_{3} e^{az} \frac{{dU_{M} (z)}}{dz} - c_{3} e^{az} U_{L} (z)} \right]\frac{\partial S}{{\partial r}} + \left[ {T_{N} (z) - c_{3} e^{az} \frac{{dU_{N} (z)}}{dz}} \right]\frac{\partial S}{{r\partial \theta }} = 0,} \\ {\left[ {T_{M} (z) - c_{3} e^{az} \frac{{dU_{M} (z)}}{dz} - c_{3} e^{az} U_{L} (z)} \right]\frac{\partial S}{{r\partial \theta }} - \left[ {T_{N} (z) - c_{3} e^{az} \frac{{dU_{N} (z)}}{dz}} \right]\frac{\partial S}{{\partial r}} = 0,} \\ {T_{L} (z) = - c_{{2}} e^{az} U_{M} (z)\lambda^{2} + c_{{1}} e^{az} \frac{{dU_{L} (z)}}{dz}.} \\ \end{array} } \right.$$

By solving Eq. (25), we can get

26 $$\frac{{dU_{L} \left( z \right)}}{dz} = \lambda^{2} \frac{{c_{2} }}{{c_{1} }}U_{M} \left( z \right) + \frac{1}{{c_{1} }}e^{ - az} T_{L} \left( z \right),$$

27 $$\frac{{dU_{M} \left( z \right)}}{dz} = - U_{L} \left( z \right) + \frac{1}{{c_{3} }}e^{ - az} T_{M} \left( z \right),$$

28 $$\frac{{dU_{N} \left( z \right)}}{dz} = \frac{1}{{c_{3} }}e^{ - az} T_{N} \left( z \right).$$

Substituting Eqs. (19)–(24) into the first relation of Eq. (1) yields

29 $$\begin{aligned} & U_{M} (z)\left[ {c_{1} \left( {\frac{{\partial^{3} S}}{{\partial r^{3} }} - \frac{{\partial^{2} S}}{{r^{3} \partial \theta^{2} }} - \frac{\partial S}{{r^{2} \partial r}} + \frac{{\partial^{2} S}}{{r\partial r^{2} }}} \right){ + }c_{2} \left( {\frac{{\partial^{3} S}}{{r^{2} \partial r\partial \theta^{2} }} - \frac{{\partial^{2} S}}{{r^{3} \partial \theta^{2} }}} \right) + 2c_{3} \left( {\frac{{\partial^{3} S}}{{r^{2} \partial \theta^{2} \partial r}} - \frac{{\partial^{2} S}}{{r^{3} \partial \theta^{2} }}} \right)} \right] \\ & \qquad + U_{N} (z)\left[ {\left( {c_{1} - c_{2} } \right)\frac{{\partial^{3} S}}{{r\partial r^{2} \partial \theta }} + c_{3} \left( {\frac{{\partial^{3} S}}{{r^{3} \partial \theta^{3} }} - \frac{{\partial^{3} S}}{{r\partial \theta \partial r^{2} }} + \frac{{\partial^{2} S}}{{r^{2} \partial \theta \partial r}}} \right)} \right] \\ & \qquad + c_{2} \frac{{dU_{L} (z)}}{dz}\frac{\partial S}{{\partial r}} + c_{3} \left( {\frac{{d^{2} U_{M} (z)}}{{dz^{2} }}\frac{\partial S}{{\partial r}} + \frac{{d^{2} U_{N} (z)}}{{dz^{2} }}\frac{\partial S}{{r\partial \theta }} + \frac{{dU_{L} (z)}}{dz}\frac{\partial S}{{\partial r}}} \right) \\ & \quad = 0. \\ \end{aligned}$$

By substituting formula (5) into Eq. (29), we have

30 $$\begin{aligned} & U_{M} (z)c_{1} \left( {\frac{{\partial^{3} S}}{{\partial r^{3} }} - \frac{{2\partial^{2} S}}{{r^{3} \partial \theta^{2} }} - \frac{\partial S}{{r^{2} \partial r}} + \frac{{\partial^{2} S}}{{r\partial r^{2} }}{ + }\frac{{\partial^{3} S}}{{r^{2} \partial \theta^{2} \partial r}}} \right) + U_{N} (z)c_{3} \left( {\frac{{\partial^{3} S}}{{r^{3} \partial \theta^{3} }} + \frac{{\partial^{3} S}}{{r\partial \theta \partial r^{2} }} + \frac{{\partial^{2} S}}{{r^{2} \partial \theta \partial r}}} \right) \\ & \quad + c_{2} \frac{{dU_{L} (z)}}{dz}\frac{\partial S}{{\partial r}} + c_{3} \left( {\frac{{d^{2} U_{M} (z)}}{{dz^{2} }}\frac{\partial S}{{\partial r}} + \frac{{d^{2} U_{N} (z)}}{{dz^{2} }}\frac{\partial S}{{r\partial \theta }} + \frac{{dU_{L} (z)}}{dz}\frac{\partial S}{{\partial r}}} \right) = 0. \\ \end{aligned}$$

Substituting Eqs. (27), (28) and (9) into Eq. (30) gives

31 $$\left[ {c_{2} e^{az} \frac{{dU_{L} (z)}}{dz}{ + }\frac{{dT_{M} (z)}}{dz} - c_{1} e^{az} \lambda^{2} U_{M} (z)} \right]\frac{\partial S}{{\partial r}}{ + }\left[ {\frac{{dT_{N} (z)}}{dz} - c_{3} e^{az} \lambda^{2} U_{N} (z)} \right]\frac{\partial S}{{r\partial \theta }} = 0.$$

Similarly, we can obtain

32 $$\left[ {c_{2} e^{az} \frac{{dU_{L} (z)}}{dz}{ + }\frac{{dT_{M} (z)}}{dz} - \lambda^{2} c_{1} e^{az} U_{M} (z)} \right]\frac{\partial S}{{r\partial \theta }} + \left[ {\frac{{dT_{N} (z)}}{dz} - \lambda^{2} c_{3} e^{az} U_{N} (z)} \right]\frac{\partial S}{{\partial r}} = 0,$$

33 $$- T_{M} (z)\lambda^{2} + \frac{{dT_{L} (z)}}{dz} = 0.$$

From Eqs. (31)–(33), we have

34 $$\frac{{dT_{L} (z)}}{dz} = \lambda^{2} T_{M} (z),$$

35 $$\frac{{dT_{M} (z)}}{dz} = \lambda^{2} \frac{{c_{1}^{2} - c_{2}^{2} }}{{c_{1} }}e^{az} U_{M} (z) - \frac{{c_{2} }}{{c_{1} }}T_{L} (z),$$

36 $$\frac{{dT_{N} (z)}}{dz} = \lambda^{2} c_{3} e^{az} U_{N} (z).$$

In this paper, the coefficient formula with *N* subscript will not appear in the following calculation when only the normal load is considered. Rewriting Eqs. (26), (27), (34) and (35) in matrix form as follows

$$\left[ {\begin{array}{*{20}c} {{{dU_{L} \left( z \right)} \mathord{\left/ {\vphantom {{dU_{L} \left( z \right)} {dz}}} \right. \kern-\nulldelimiterspace} {dz}}} \\ {{{dU_{M} \left( z \right)} \mathord{\left/ {\vphantom {{dU_{M} \left( z \right)} {dz}}} \right. \kern-\nulldelimiterspace} {dz}}} \\ {{{dT_{L} \left( z \right)} \mathord{\left/ {\vphantom {{dT_{L} \left( z \right)} {dz}}} \right. \kern-\nulldelimiterspace} {dz}}} \\ {{{dT_{M} \left( z \right)} \mathord{\left/ {\vphantom {{dT_{M} \left( z \right)} {dz}}} \right. \kern-\nulldelimiterspace} {dz}}} \\ \end{array} } \right]\; = \left[ {\begin{array}{*{20}c} 0 & {\lambda^{2} \frac{{c_{2} }}{{c_{1} }}} & {\frac{1}{{c_{1} }}e^{ - az} } & 0 \\ { - 1} & 0 & 0 & {\frac{1}{{c_{3} }}e^{ - az} } \\ 0 & 0 & 0 & {\lambda^{2} } \\ 0 & {\lambda^{2} \frac{{c_{1}^{2} - c_{2}^{2} }}{{c_{1} }}e^{az} } & { - \frac{{c_{2} }}{{c_{1} }}} & 0 \\ \end{array} } \right]\left[ {\begin{array}{*{20}c} {U_{L} \left( z \right)} \\ {U_{M} \left( z \right)} \\ {T_{L} \left( z \right)} \\ {T_{M} \left( z \right)} \\ \end{array} } \right].$$

Rewriting the former formula gives

$$\left[ {E^{*} } \right]_{,z} = \left[ {\begin{array}{*{20}c} {{{dU_{L} \left( z \right)} \mathord{\left/ {\vphantom {{dU_{L} \left( z \right)} {dz}}} \right. \kern-\nulldelimiterspace} {dz}}} \\ {{{\lambda dU_{M} \left( z \right)} \mathord{\left/ {\vphantom {{\lambda dU_{M} \left( z \right)} {dz}}} \right. \kern-\nulldelimiterspace} {dz}}} \\ {{{dT_{L} \left( z \right)e^{ - az} } \mathord{\left/ {\vphantom {{dT_{L} \left( z \right)e^{ - az} } {\lambda dz}}} \right. \kern-\nulldelimiterspace} {\lambda dz}}} \\ {{{dT_{M} \left( z \right)e^{ - az} } \mathord{\left/ {\vphantom {{dT_{M} \left( z \right)e^{ - az} } {dz}}} \right. \kern-\nulldelimiterspace} {dz}}} \\ \end{array} } \right]\; = \lambda \left[ {\begin{array}{*{20}c} {0} & {\frac{{c_{2} }}{{c_{1} }}} & {\frac{1}{{c_{1} }}} & 0 \\ { - 1} & 0 & 0 & {\frac{1}{{c_{3} }}} \\ 0 & 0 & { - \frac{a}{\lambda }} & 1 \\ 0 & {\frac{{c_{1}^{2} - c_{2}^{2} }}{{c_{1} }}} & { - \frac{{c_{2} }}{{c_{1} }}} & { - \frac{a}{\lambda }} \\ \end{array} } \right]\left[ {\begin{array}{*{20}c} {U_{L} \left( z \right)} \\ {\lambda U_{M} \left( z \right)} \\ {\frac{{T_{L} \left( z \right)e^{ - az} }}{\lambda }} \\ {T_{M} \left( z \right)e^{ - az} } \\ \end{array} } \right].$$

In this case, with the help of the vector function system in cylindrical coordinates, the partial differential equations are transformed into the following ordinary differential equations as

37 $$\left[ {\frac{{dE^{*} }}{dz}} \right] = \lambda \left[ A \right]\left[ {E^{*} } \right],$$

with $$\left[ {E^{*} } \right] = \left[ P \right]\left[ E \right],$$ where

$$\left[ P \right] = \left[ {\begin{array}{*{20}c} 1 & 0 & 0 & 0 \\ 0 & 1 & 0 & 0 \\ 0 & 0 & {e^{ - az} } & 0 \\ 0 & 0 & 0 & {e^{ - az} } \\ \end{array} } \right],\,\left[ E \right] = \left[ {\begin{array}{*{20}c} {U_{L} \left( z \right)} \\ {\lambda U_{M} \left( z \right)} \\ {{{T_{L} \left( z \right)} \mathord{\left/ {\vphantom {{T_{L} \left( z \right)} \lambda }} \right. \kern-\nulldelimiterspace} \lambda }} \\ {T_{M} \left( z \right)} \\ \end{array} } \right].$$

The vector function of the stress boundary condition at the structural surface layer $z = 0$ in the cylindrical coordinate system is expressed as^33^

$${\varvec{P}}\left( {r,\theta ,z} \right) = \int\limits_{0}^{\infty } {\left[ {P_{L} \left( {\lambda ,z} \right){\varvec{L}}\left( {r,\theta } \right) + P_{M} \left( {\lambda ,z} \right){\varvec{M}}\left( {r,\theta } \right)} \right]\lambda d\lambda } ,$$

The expansion coefficient is expressed as follows

$$P_{L} (\lambda ,z) = \int\limits_{0}^{2\pi } {\int\limits_{0}^{\infty } {{\varvec{P}}(r,\theta ,z) \cdot } } \overline{\user2{L}}(r,\theta )rdrd\theta ,\;\;P_{M} (\lambda ,z) = \frac{1}{{\lambda^{2} }}\int\limits_{0}^{2\pi } {\int\limits_{0}^{\infty } {{\varvec{P}}(r,\theta ,z) \cdot } } \overline{\user2{M}}(r,\theta )rdrd\theta .$$

Thus, the stress boundary conditions become

$$P_{L} \left( {\lambda ,0} \right) = \int\limits_{0}^{2\pi } {\int\limits_{0}^{\infty } {\left( {\sigma_{zz} \left( {r,\theta ,0} \right){\varvec{e}}_{z} } \right) \cdot } } \overline{\user2{L}}\left( {r,\theta } \right)rdrd\theta = - \sqrt {2\pi } \gamma_{lv} \left( {RJ_{0} \left( {\lambda R} \right) - \frac{{2J_{1} \left( {\lambda R} \right)}}{\lambda }} \right),$$

$$P_{M} \left( {\lambda ,0} \right) = \frac{1}{{\lambda^{2} }}\int\limits_{0}^{2\pi } {\int\limits_{0}^{\infty } {\left( {\sigma_{zz} \left( {r,\theta ,0} \right){\varvec{e}}_{z} } \right) \cdot } } \overline{\user2{M}}\left( {r,\theta } \right)rdrd\theta = 0.$$

Then the coefficients of the surface displacement and stress boundary conditions under the vector function system can be expressed as

38 $$U_{L} (z_{0} ) = U_{L} (0),\;\lambda U_{M} (z_{0} ) = \lambda U_{M} (0),\;T_{M} (z_{0} ) = T_{M} (0) = 0,$$

39 $$\frac{{T_{L} (z_{0} )}}{\lambda } = \frac{{P_{L} (\lambda ,0)}}{\lambda } = - \frac{{\sqrt {2\pi } \gamma_{lv} }}{\lambda }\left( {RJ_{0} (\lambda R) - \frac{{2J_{1} (\lambda R)}}{\lambda }} \right) \triangleq P_{L0} .$$

## Solutions of the model

For Eq. (37), let $\nu_{i}$ be the eigenvalue of the matrix $\left[ A \right]$, and the matrix $\left[ B \right]$ be formed by the eigenvector corresponding to the eigenvalue, and its general solution is^33^

40 $$\left[ {E^{*} } \right] = \left[ {Z\left( z \right)} \right]\left[ K \right],$$

where $\left[ K \right]$ is $4 \times 1$ coefficient matrix, and its value is determined by the boundary conditions or interface conditions. Also, we have

41 $$\left[ {Z\left( z \right)} \right] = \left[ B \right]\left\langle {e^{\lambda \nu^{ *}z} } \right\rangle ,$$

with

$$\left\langle {e^{\lambda \nu ^{*}z} } \right\rangle {\text{ = diag}}\left( {e^{{\lambda \nu_{1} z}} ,e^{{\lambda \nu_{2} z}} ,e^{{\lambda \nu_{3} z}} ,e^{{\lambda \nu_{4} z}} } \right).$$

The transfer relation of layer $k$ coefficient vector $\left[ E \right]$ connects the value at global coordinate $z_{k - 1} \left( {\xi = 0} \right)$ with the value at $z_{k} \left( {\xi = h_{k} } \right)$, and the relation is^33^

42 $$\left[ {E\left( {z_{k - 1} } \right)} \right] = \left[ {a_{k} } \right]\left[ {E\left( {z_{k} } \right)} \right],$$

where

$$\left[ {a_{k} } \right] = \left[ B \right]\left\langle {e^{{ - \lambda \nu_{k}^{*} h_{k} }} } \right\rangle \left[ B \right]^{ - 1} \left[ P \right],$$

with

$$\left\langle {e^{{ - \lambda \nu_{k}^{*} h_{k} }} } \right\rangle {\text{ = diag}}\left( {e^{{ - \lambda \nu_{k1} h_{k} }} ,e^{{ - \lambda \nu_{k2} h_{k} }} ,e^{{ - \lambda \nu_{k3} h_{k} }} ,e^{{ - \lambda \nu_{k4} h_{k} }} } \right).$$

Next, we consider the wetting phenomenon of the substrate with a rigid layer and semi-infinite space.

1. If the bottom boundary is a rigid layer, then the displacement of the bottom surface of the substrate is zero, so the transfer relation of the layered structure is
  43 $$\left[ {E\left( {z_{0} } \right)} \right] = \left[ {a_{1} } \right]\left[ {a_{2} } \right] \cdots \left[ {a_{n} } \right]\left[ {E\left( {z_{n} } \right)} \right].$$

Now the displacement coefficient of $\left[ {E(z_{n} )} \right]$ at the interface $z = z_{n}$ on the rigid layer is $U_{L} \left( {z_{n} } \right) = U_{M} \left( {z_{n} } \right) = 0$, and the boundary conditions at the surface $z = z_{0}$ are shown in Eqs. (38) and (39), then Eq. (43) becomes

44 $$\left[ {\begin{array}{*{20}c} {U_{L} \left( 0 \right)} \\ {\lambda U_{M} \left( 0 \right)} \\ {P_{L0} } \\ 0 \\ \end{array} } \right] = \left[ {a_{{\text{total}}} } \right]\left[ {\begin{array}{*{20}c} 0 \\ 0 \\ {{{T_{L} \left( {z_{n} } \right)} \mathord{\left/ {\vphantom {{T_{L} \left( {z_{n} } \right)} \lambda }} \right. \kern-\nulldelimiterspace} \lambda }} \\ {T_{M} \left( {z_{n} } \right)} \\ \end{array} } \right]$$

with $\left[ {a_{1} } \right]\left[ {a_{2} } \right] \cdots \left[ {a_{n} } \right] = \left[ {a_{{\text{total}}} } \right]{ = (}a_{l,m} {)}_{4 \times 4} ,\;\left( {l,m = 1,2,3,4} \right)$. Therefore, Eq. (44) is a system of quaternion linear equations about unknown quantity $U_{L} (0),U_{M} (0),T_{L} (z_{n} ),T_{M} (z_{n} )$, and we can obtain

$$T_{L} \left( {z_{n} } \right) = \frac{{\lambda a_{44} P_{L0} }}{{a_{33} a_{44} - a_{43} a_{34} }},\;\;T_{M} \left( {z_{n} } \right) = \frac{{ - a_{43} P_{L0} }}{{a_{33} a_{44} - a_{43} a_{34} }}.$$

$$U_{L} \left( 0 \right) = \frac{{\left( {a_{13} a_{44} - a_{14} a_{43} } \right)P_{L0} }}{{a_{33} a_{44} - a_{43} a_{34} }},\;\;U_{M} \left( 0 \right) = \frac{{\left( {a_{23} a_{44} - a_{24} a_{43} } \right)P_{L0} }}{{\lambda \left( {a_{33} a_{44} - a_{43} a_{34} } \right)}}.$$

In this way, the displacement coefficients $U_{L} (0),U_{M} (0)$ of $\left[ {E(z_{0} )} \right]$ at $z = z_{0}$ and the stress coefficients $T_{L} (z_{n} ),T_{M} (z_{n} )$ of $\left[ {E(z_{n} )} \right]$ at $z = z_{n}$ have been solved, so the displacement and stress of the whole structure can be obtained.

According to formula (12), the normal displacement of the surface deformation of the substrate is

$$\begin{aligned} u_{z} \left( {r,0} \right) & = \frac{1}{{\sqrt {2\pi } }}\int_{0}^{ + \infty } {U_{L} \left( 0 \right)} J_{0} \left( {\lambda r} \right)\lambda d\lambda \\ & = R\int_{0}^{ + \infty } {C_{uz} \left( \lambda \right)J_{0} \left( {\lambda R} \right)} J_{0} \left( {\lambda r} \right)d\lambda - 2\int_{0}^{ + \infty } {\frac{1}{\lambda }C_{uz} \left( \lambda \right)J_{1} \left( {\lambda R} \right)} J_{0} \left( {\lambda r} \right)d\lambda , \\ \end{aligned}$$

with

$$C_{uz} \left( \lambda \right) = - \frac{{a_{13} a_{44} - a_{14} a_{43} }}{{a_{33} a_{44} - a_{43} a_{34} }}\gamma_{lv} .$$

The radial displacement of the surface deformation is

$$\begin{aligned} u_{r} \left( {r,0} \right) & = \frac{ - 1}{{\sqrt {2\pi } }}\int_{0}^{ + \infty } {U_{M} \left( 0 \right)} J_{1} \left( {\lambda r} \right)\lambda^{2} d\lambda \\ & = R\int_{0}^{ + \infty } {C_{ur} \left( \lambda \right)J_{0} \left( {\lambda R} \right)} J_{1} \left( {\lambda r} \right)d\lambda - 2\int_{0}^{ + \infty } {\frac{1}{\lambda }C_{ur} \left( \lambda \right)J_{1} \left( {\lambda R} \right)} J_{1} \left( {\lambda r} \right)d\lambda , \\ \end{aligned}$$

with

$$C_{ur} \left( \lambda \right) = \frac{{a_{23} a_{44} - a_{24} a_{43} }}{{a_{33} a_{44} - a_{43} a_{34} }}\gamma_{lv} .$$

According to formula (13), the normal stress on the bottom of the substrate is

$$\begin{aligned} \sigma_{zz} \left( {r,z_{n} } \right) & = \frac{1}{{\sqrt {2\pi } }}\int_{0}^{ + \infty } {T_{L} \left( {z_{n} } \right)} J_{0} \left( {\lambda r} \right)\lambda d\lambda \\ & = R\int_{0}^{ + \infty } {\lambda C_{\sigma z} \left( \lambda \right)J_{0} \left( {\lambda R} \right)} J_{0} \left( {\lambda r} \right)d\lambda - 2\int_{0}^{ + \infty } {C_{\sigma z} \left( \lambda \right)J_{1} \left( {\lambda R} \right)} J_{0} \left( {\lambda r} \right)d\lambda , \\ \end{aligned}$$

with

$$C_{\sigma z} \left( \lambda \right) = \frac{{ - a_{44} \gamma_{lv} }}{{a_{33} a_{44} - a_{43} a_{34} }}.$$

The radial shear stress on the bottom of the substrate is

$$\begin{aligned} \sigma_{rz} \left( {r,z_{n} } \right) & = \frac{ - 1}{{\sqrt {2\pi } }}\int_{0}^{ + \infty } {T_{M} \left( {z_{n} } \right)} J_{1} \left( {\lambda r} \right)\lambda^{2} d\lambda \\ & = R\int_{0}^{ + \infty } {\lambda C_{\sigma r} \left( \lambda \right)J_{0} \left( {\lambda R} \right)} J_{1} \left( {\lambda r} \right)d\lambda - 2\int_{0}^{ + \infty } {C_{\sigma r} \left( \lambda \right)J_{1} \left( {\lambda R} \right)} J_{1} \left( {\lambda r} \right)d\lambda , \\ \end{aligned}$$

with

$$C_{\sigma r} \left( \lambda \right) = \frac{{ - a_{43} \gamma_{lv} }}{{a_{33} a_{44} - a_{43} a_{34} }}.$$

Since $$\left[ {E(z_{0} )} \right]$$ has been solved, Eq. (42) becomes a set of quaternion linear equations about the expansion coefficients $$U_{L} (z_{k} ),$$$$U_{M} (z_{k} ),$$$$T_{L} (z_{k} ),$$$$T_{M} (z_{k} )$$ of the displacement and stress in the *k*-layer. Thus, the normal displacement of each inter-layer deformation can be obtained as follows

$$u_{z} \left( {r,z_{k} } \right) = \int_{0}^{ + \infty } {U_{L} \left( {z_{k} } \right)S\left( {r,\lambda } \right)} \lambda d\lambda = \frac{1}{{\sqrt {2\pi } }}\int_{0}^{ + \infty } {U_{L} \left( {z_{k} } \right)} J_{0} \left( {\lambda r} \right)\lambda d\lambda .$$

The radial displacement of the inter-layer is

$$u_{r} \left( {r,z_{k} } \right) = \int_{0}^{ + \infty } {U_{M} \left( {z_{k} } \right)\frac{{\partial S\left( {r,\lambda } \right)}}{\partial r}} \lambda d\lambda = \frac{ - 1}{{\sqrt {2\pi } }}\int_{0}^{ + \infty } {U_{M} \left( {z_{k} } \right)} J_{1} \left( {\lambda r} \right)\lambda^{2} d\lambda .$$

According to the expansion formula of stress, the normal stress of the inter-layer is

$$\sigma_{zz} \left( {r,z_{k} } \right) = \int_{0}^{ + \infty } {T_{L} \left( {z_{k} } \right)S\left( {r,\lambda } \right)} \lambda d\lambda = \frac{1}{{\sqrt {2\pi } }}\int_{0}^{ + \infty } {T_{L} \left( {z_{k} } \right)} J_{0} \left( {\lambda r} \right)\lambda d\lambda .$$

The radial shear stress of the inter-layer is

$$\sigma_{rz} \left( {r,z_{k} } \right) = \int_{0}^{ + \infty } {T_{M} \left( {z_{k} } \right)\frac{{\partial S\left( {r,\lambda } \right)}}{\partial r}} \lambda d\lambda = \frac{ - 1}{{\sqrt {2\pi } }}\int_{0}^{ + \infty } {T_{M} \left( {z_{k} } \right)} J_{1} \left( {\lambda r} \right)\lambda^{2} d\lambda .$$

(2) If the bottom boundary is a semi-infinite space, the displacement at infinity is zero. The overall transfer relation is
  45 $$\left[ {E\left( {z_{0} } \right)} \right] = \left[ {a_{1} } \right]\left[ {a_{2} } \right] \cdots \left[ {a_{n} } \right]\left[ {E^{*} \left( {z_{n} } \right)} \right] = \left[ {a_{1} } \right] \cdots \left[ {a_{n} } \right]\left[ {B_{n} } \right]\left\langle {e^{{\lambda \nu_{n}^{*} h_{n} }} } \right\rangle \left[ K \right],$$
  where $\left[ {a_{k} } \right]$ is the transfer matrix corresponding to *k*-layer.

In a continuous homogeneous semi-infinite space at $z = z_{0}$, the displacement at infinity is zero, so the two coefficients corresponding to the displacement in matrix $\left[ K \right]$ are zero, and the coefficients corresponding to the stress are $K_{31} ,K_{41}$. As the traction force exerted by the droplet on the substrate at the surface is solved, the coefficient containing $T_{L} ,T_{M}$ in $\left[ {E(z_{0} )} \right]$ will be determined, then Eq. (45) becomes

46 $$\left[ {\begin{array}{*{20}c} {U_{L1} \left( 0 \right)} \\ {\lambda U_{M1} \left( 0 \right)} \\ {P_{L0} } \\ 0 \\ \end{array} } \right] = \left[ {b_{{\text{total}}} } \right]\left[ {\begin{array}{*{20}c} 0 \\ 0 \\ {K_{31} } \\ {K_{41} } \\ \end{array} } \right],$$

where

$$\left[ {b_{{\text{total}}} } \right] = \left[ {a_{1} } \right]\left[ {a_{2} } \right] \cdots \left[ {a_{n} } \right]\left[ {B_{n} } \right]\left\langle {e^{{\lambda \nu_{n}^{*} h_{n} }} } \right\rangle = \left( {b_{l,m} } \right)_{4 \times 4} ,\;\;\left( {l,m = 1,2,3,4} \right).$$

Equation (46) is also a system of quaternion linear equations, which can be easily solved as

$$K_{31} = \frac{{b_{44} P_{L0} }}{{b_{33} b_{44} - b_{43} b_{34} }},\;\;K_{41} = \frac{{ - b_{43} P_{L0} }}{{b_{33} b_{44} - b_{43} b_{34} }}.$$

$$U_{L1} (0) = \frac{{\left( {b_{13} b_{44} - b_{14} b_{43} } \right)P_{L0} }}{{b_{33} b_{44} - b_{43} b_{34} }},\;\;U_{M1} (0) = \frac{{\left( {b_{23} b_{44} - b_{24} b_{43} } \right)P_{L0} }}{{\lambda \left( {b_{33} b_{44} - b_{43} b_{34} } \right)}}.$$

At this point, the coefficients of the displacement in $\left[ {E(z_{0} )} \right]$ and the stress in matrix $\left[ K \right]$ are obtained, the normal displacement of substrate surface deformation is

$$\begin{aligned} u_{z} \left( {r,0} \right) & = \frac{1}{{\sqrt {2\pi } }}\int_{0}^{ + \infty } {U_{L1} \left( 0 \right)} J_{0} \left( {\lambda r} \right)\lambda d\lambda \\ & = R\int_{0}^{ + \infty } {C_{uz1} \left( \lambda \right)J_{0} \left( {\lambda R} \right)} J_{0} \left( {\lambda r} \right)d\lambda - 2\int_{0}^{ + \infty } {\frac{1}{\lambda }C_{uz1} \left( \lambda \right)J_{1} \left( {\lambda R} \right)} J_{0} \left( {\lambda r} \right)d\lambda , \\ \end{aligned}$$

with

$$C_{uz1} \left( \lambda \right) = - \frac{{b_{13} b_{44} - b_{14} b_{43} }}{{b_{33} b_{44} - b_{43} b_{34} }}\gamma_{lv} .$$

The radial displacement of the surface deformation is

$$\begin{aligned} u_{r} \left( {r,0} \right) & = \frac{ - 1}{{\sqrt {2\pi } }}\int_{0}^{ + \infty } {U_{M1} \left( 0 \right)} J_{1} \left( {\lambda r} \right)\lambda^{2} d\lambda \\ & = R\int_{0}^{ + \infty } {C_{ur1} \left( \lambda \right)J_{0} \left( {\lambda R} \right)} J_{1} \left( {\lambda r} \right)d\lambda - 2\int_{0}^{ + \infty } {\frac{1}{\lambda }C_{ur1} \left( \lambda \right)J_{1} \left( {\lambda R} \right)} J_{1} \left( {\lambda r} \right)d\lambda , \\ \end{aligned}$$

with

$$C_{ur1} \left( \lambda \right) = \frac{{b_{23} b_{44} - b_{24} b_{43} }}{{b_{33} b_{44} - b_{43} b_{34} }}\gamma_{lv} .$$

From Eqs. (40) and (41), we can obtain

$$\left[ {\begin{array}{*{20}c} {U_{L1} \left( {z_{n} } \right)} \\ {\lambda U_{M1} \left( {z_{n} } \right)} \\ {{{T_{L1} \left( {z_{n} } \right)} \mathord{\left/ {\vphantom {{T_{L1} \left( {z_{n} } \right)} \lambda }} \right. \kern-\nulldelimiterspace} \lambda }} \\ {T_{M1} \left( {z_{n} } \right)} \\ \end{array} } \right] = \left[ {B_{n} } \right]\left\langle {e^{{\lambda \nu_{n}^{*} h_{n} }} } \right\rangle \left[ K \right].$$

The normal stress on the bottom of the substrate is

$$\sigma_{zz} \left( {r,z_{n} } \right) = \frac{1}{{\sqrt {2\pi } }}\int_{0}^{ + \infty } {T_{L1} \left( {z_{n} } \right)} J_{0} \left( {\lambda r} \right)\lambda d\lambda .$$

The radial shear stress of the bottom surface of the substrate is

$$\sigma_{rz} \left( {r,z_{n} } \right) = \frac{ - 1}{{\sqrt {2\pi } }}\int_{0}^{ + \infty } {T_{M1} \left( {z_{n} } \right)} J_{1} \left( {\lambda r} \right)\lambda^{2} d\lambda .$$

The solution of displacement and stress between layers is similar to that of rigid layer, and the expressions of the displacement and stress are as follows.

The normal displacement of the inter-layer surface is

$$u_{z} \left( {r,z_{k} } \right) = \int_{0}^{ + \infty } {U_{L1} \left( {z_{k} } \right)S\left( {r,\lambda } \right)} \lambda d\lambda = \frac{1}{{\sqrt {2\pi } }}\int_{0}^{ + \infty } {U_{L1} \left( {z_{k} } \right)} J_{0} \left( {\lambda r} \right)\lambda d\lambda .$$

The radial displacement of the inter-layer is

$$u_{r} \left( {r,z_{k} } \right) = \int_{0}^{ + \infty } {U_{M1} \left( {z_{k} } \right)\frac{{\partial S\left( {r,\lambda } \right)}}{\partial r}} \lambda d\lambda = \frac{ - 1}{{\sqrt {2\pi } }}\int_{0}^{ + \infty } {U_{M1} \left( {z_{k} } \right)} J_{1} \left( {\lambda r} \right)\lambda^{2} d\lambda .$$

The normal stress of the inter-layer is

$$\sigma_{zz} \left( {r,z_{k} } \right) = \int_{0}^{ + \infty } {T_{L1} \left( {z_{k} } \right)S\left( {r,\lambda } \right)} \lambda d\lambda = \frac{1}{{\sqrt {2\pi } }}\int_{0}^{ + \infty } {T_{L1} \left( {z_{k} } \right)} J_{0} \left( {\lambda r} \right)\lambda d\lambda .$$

The radial shear stress of the inter-layer is

$$\sigma_{rz} \left( {r,z_{k} } \right) = \int_{0}^{ + \infty } {T_{M1} \left( {z_{k} } \right)\frac{{\partial S\left( {r,\lambda } \right)}}{\partial r}} \lambda d\lambda = \frac{ - 1}{{\sqrt {2\pi } }}\int_{0}^{ + \infty } {T_{M1} \left( {z_{k} } \right)} J_{1} \left( {\lambda r} \right)\lambda^{2} d\lambda .$$

As a result, we have solved the problems of deformation and stress on the surface, the bottom and the inter-layer, respectively. For the displacement and stress of any point in the field, the layered technology is implemented to discuss. Taking the *k*-layer as an example, the local coordinate system $O_{1} - r - \xi$ is established. From the point to be solved, the layer is divided into two layers with thickness $h_{k1} ,h_{k2}$, respectively, as shown in Fig. 1. In this case, the boundary conditions of the substrate surface and the bottom are given, so it is easy to establish the transfer relation from the point to the last layer.

1. The bottom boundary is rigid layer, and the transfer relation is
  $$\left[ {E\left( {z_{hk2} } \right)} \right] = \left[ {a_{hk2} } \right]\left[ {a_{k + 1} } \right] \cdots \left[ {a_{n} } \right]\left[ {E\left( {z_{n} } \right)} \right].$$

Using the bottom boundary conditions, we can easily obtain the expansion coefficients $$U_{L} (z_{hk2} ),$$ $$U_{M} (z_{hk2} ),$$$$T_{L} (z_{hk2} ),$$ and $$T_{M} (z_{hk2} )$$ of the displacement and stress.

The normal displacement is

$$u_{z} \left( {r,z_{hk2} } \right) = \frac{1}{{\sqrt {2\pi } }}\int_{0}^{ + \infty } {U_{L} \left( {z_{hk2} } \right)} J_{0} \left( {\lambda r} \right)\lambda d\lambda .$$

The radial displacement is expressed as

$$u_{r} \left( {r,z_{hk2} } \right) = \frac{ - 1}{{\sqrt {2\pi } }}\int_{0}^{ + \infty } {U_{M} \left( {z_{hk2} } \right)} J_{1} \left( {\lambda r} \right)\lambda^{2} d\lambda .$$

The normal stress has the form as follow

$$\sigma_{zz} \left( {r,z_{hk2} } \right) = \frac{1}{{\sqrt {2\pi } }}\int_{0}^{ + \infty } {T_{L} \left( {z_{hk2} } \right)} J_{0} \left( {\lambda r} \right)\lambda d\lambda .$$

The radial shear stress is

$$\sigma_{rz} \left( {r,z_{hk2} } \right) = \frac{ - 1}{{\sqrt {2\pi } }}\int_{0}^{ + \infty } {T_{M} \left( {z_{hk2} } \right)} J_{1} \left( {\lambda r} \right)\lambda^{2} d\lambda .$$

(2) The bottom boundary is semi-infinite space, and the transfer relation is
  $$\left[ {E\left( {z_{hk2} } \right)} \right] = \left[ {a_{hk2} } \right]\left[ {a_{k + 1} } \right] \cdots \left[ {a_{n} } \right]\left[ {B_{n} } \right]\left\langle {e^{{\lambda \nu_{n}^{*} h_{n} }} } \right\rangle \left[ K \right].$$

It is similar to the process of case (1), the stress and displacement can be easily obtained by using the bottom boundary condition, so we will not repeat it here.

## Numerical results and discussion

**Figure 2:**
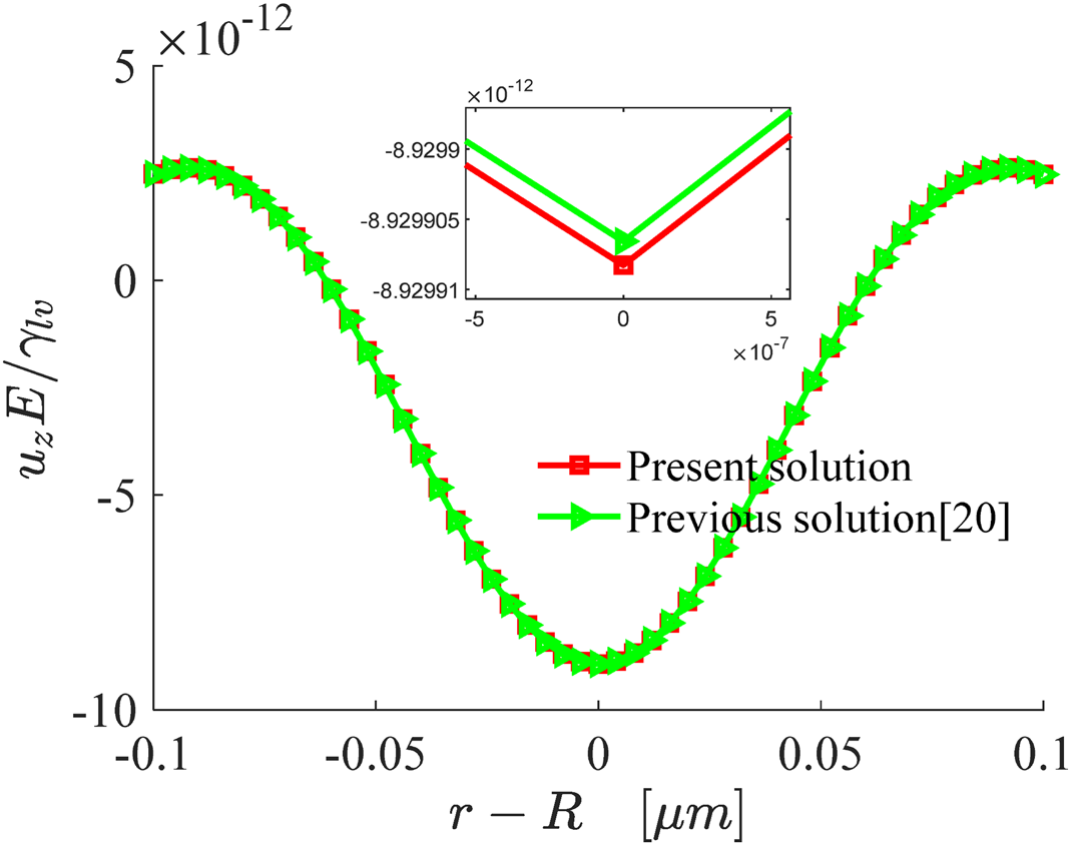
Comparison of the normal displacement in our works with that in Ref.^20^ (the small image is the result of partial enlarged drawing).

Taking the radius of droplet $R = 200\;{\upmu m}$, thickness of substrate $h = 50\;{\upmu m}$, elastic modulus of substrate $E = 3\;\text{kPa}$, Poisson's ratio $\nu = 0.48$ and gradient index $a = 0$, Fig. 2 shows the comparisons between the normal displacement of our results and those in Ref.^20^. It can be seen that the deformation displacement of a single-layer structure in this work is in good agreement with that in Ref.^20^, and the error range is within $10^{ - 7}$, which shows that the model established in this paper is correct.

Generally, the gradient indexes of different layers are different. For the convenience of discussion, we assume that the gradient index of each layer is *a*. When the droplet radius *R* and the substrate thickness *h* satisfy ${R \mathord{\left/ {\vphantom {R h}} \right. \kern-\nulldelimiterspace} h} = {\rm O}(10)$, the numerical results of a single-layer structure and double layer structure are obtained.

(I) The single layer structure is wetted by liquid droplets, and let $R = 200\;{\upmu m}$, $a = 2000$, $\gamma_{lv} = 0.05\;\text{Nm}^{ - 1}$, $E_{1} = 3\;\text{kPa}$, and $\nu_{1} = 0.48$. The displacement and stress with the thickness of the substrate are as follows.

**Figure 3:**
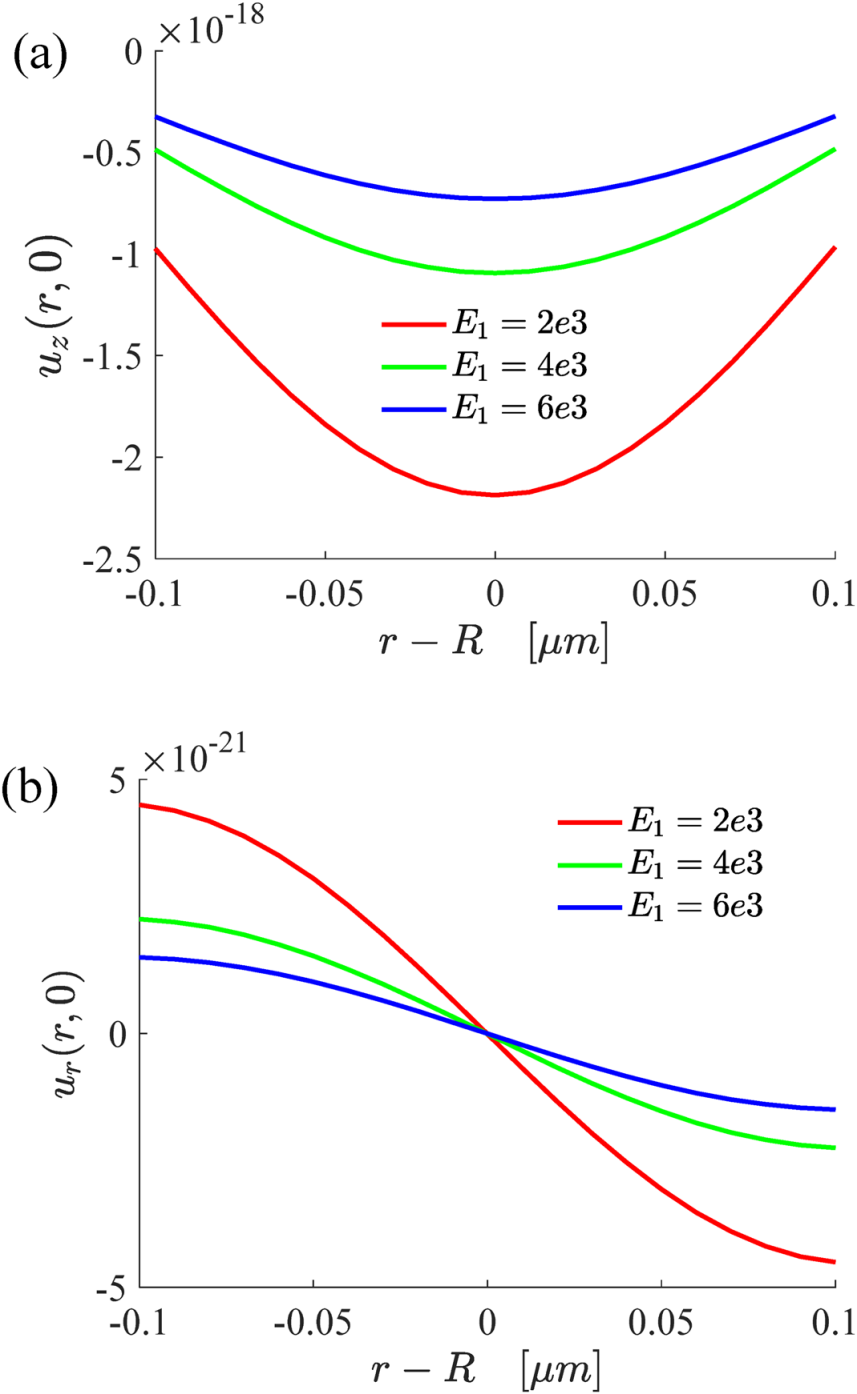
The variations of the normal (**a**) and radial (**b**) displacements with elastic modulus of the substrate.

Figure 3 shows the variations of substrate surface deformation with modulus. It can be seen that the normal and radial deformations of the substrate decrease with the increase of the elastic modulus of the substrate, which is consistent with the experimental results.

**Figure 4:**
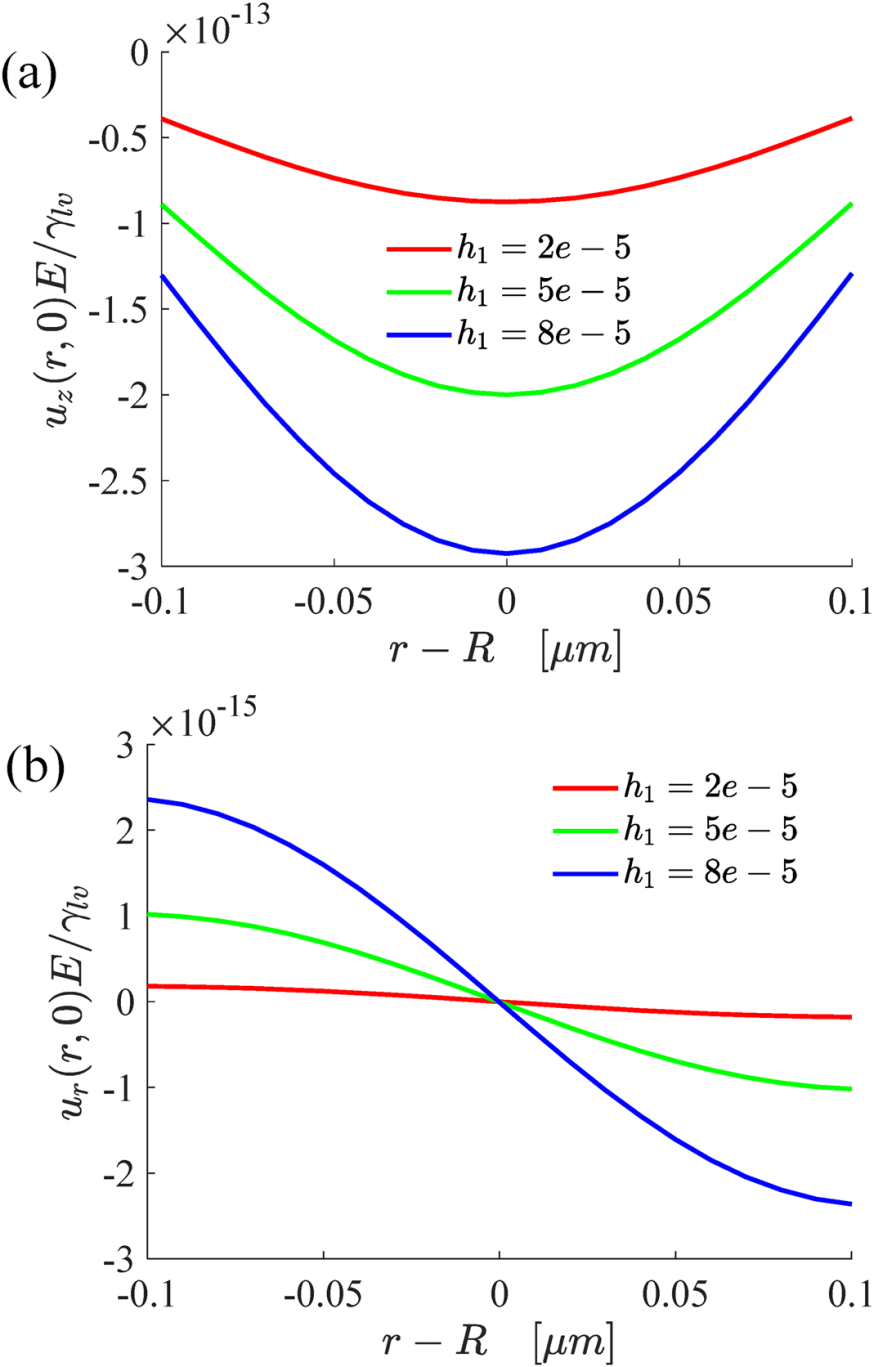
The variations of the normal (**a**) and radial (**b**) displacements of the surface deformation.

Figure 4 shows the variations of the normal and radial displacements of the single-layer structure surface deformation with the contact radius and substrate thickness. The results indicate that the normal displacement increases with the increase of substrate thickness, and the change range of wetting ridge also increases. Similarly, with the increase of substrate thickness, the radial deformation is becoming ever more larger. However, compared with the normal deformation, the radial deformation is very weak, so the normal displacement can well describe the surface profile of the substrate.

**Figure 5:**
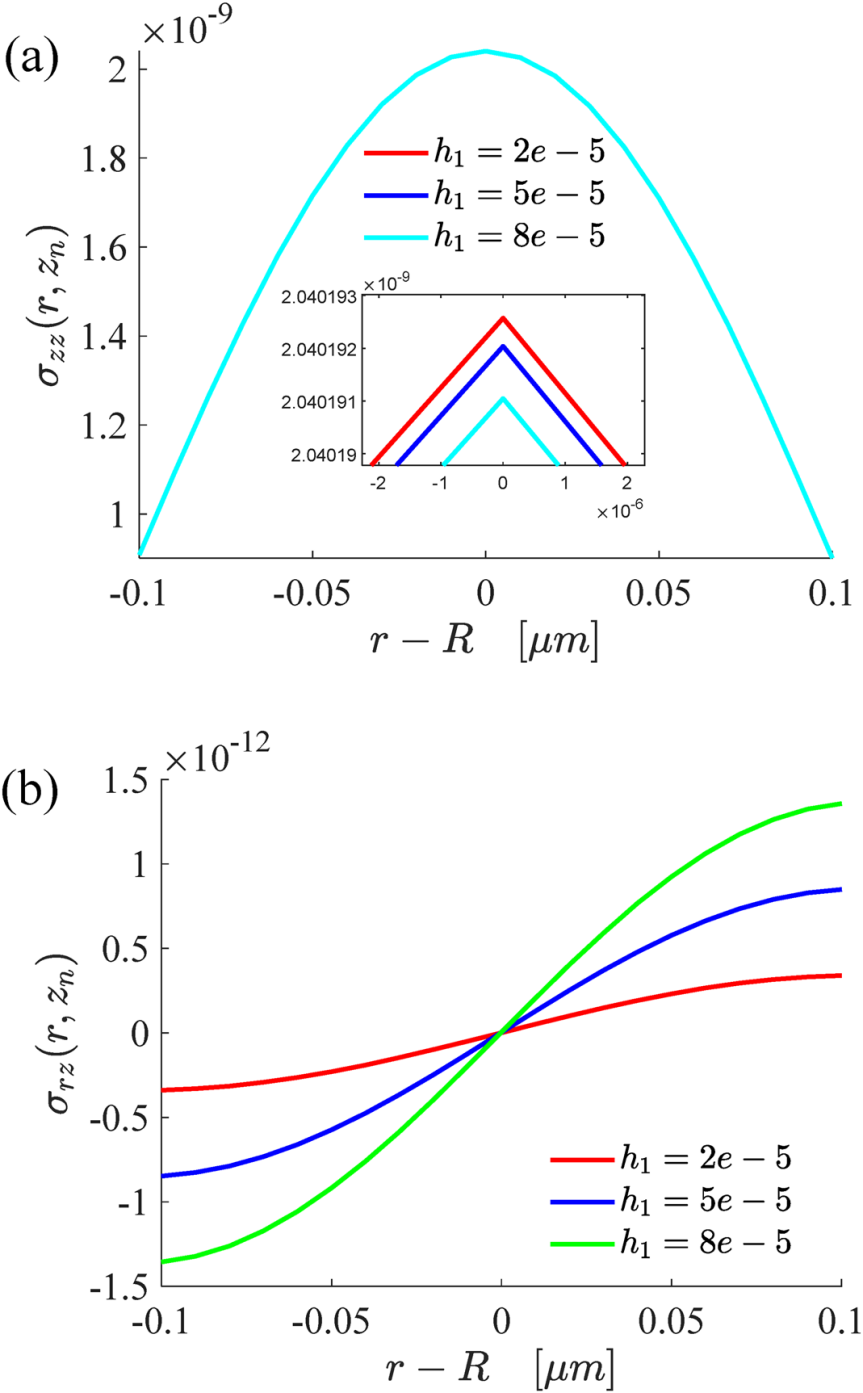
The variations of the normal stress (**a**) and radial shear stress (**b**) on the bottom surface of the substrate.

Figure 5 demonstrates the variations of the normal stress and tangential normal stress of the bottom surface with contact radius and substrate thickness under single layer structure. It shows that the substrate thickness has little effect on normal stress, and it can be seen from local enlargement that the whole normal stress increases with the decrease of the substrate thickness. On the contrary, the shear stress decreases with the decrease of substrate thickness. Comparing Fig. 4 with Fig. 5, it is found that the changes of radial displacement and shear stress show opposite trends.

(II) The droplet wets the bilayer structure, and let $R = 200\;{\upmu \text{m}}$, $a = 20$, $h_{1} = 20\;{\upmu \text{m}}$, $E_{1} = 2\;\text{kPa}$, $\nu_{1} = 0.42$, $h_{2} = 60\;{\upmu \text{m}}$, $E_{2} = 4\;\text{kPa}$, $\nu_{2} = 0.44$, and $\gamma_{lv} = 0.05\;\text{Nm}^{ - 1}$. The following numerical results are obtained.

**Figure 6:**
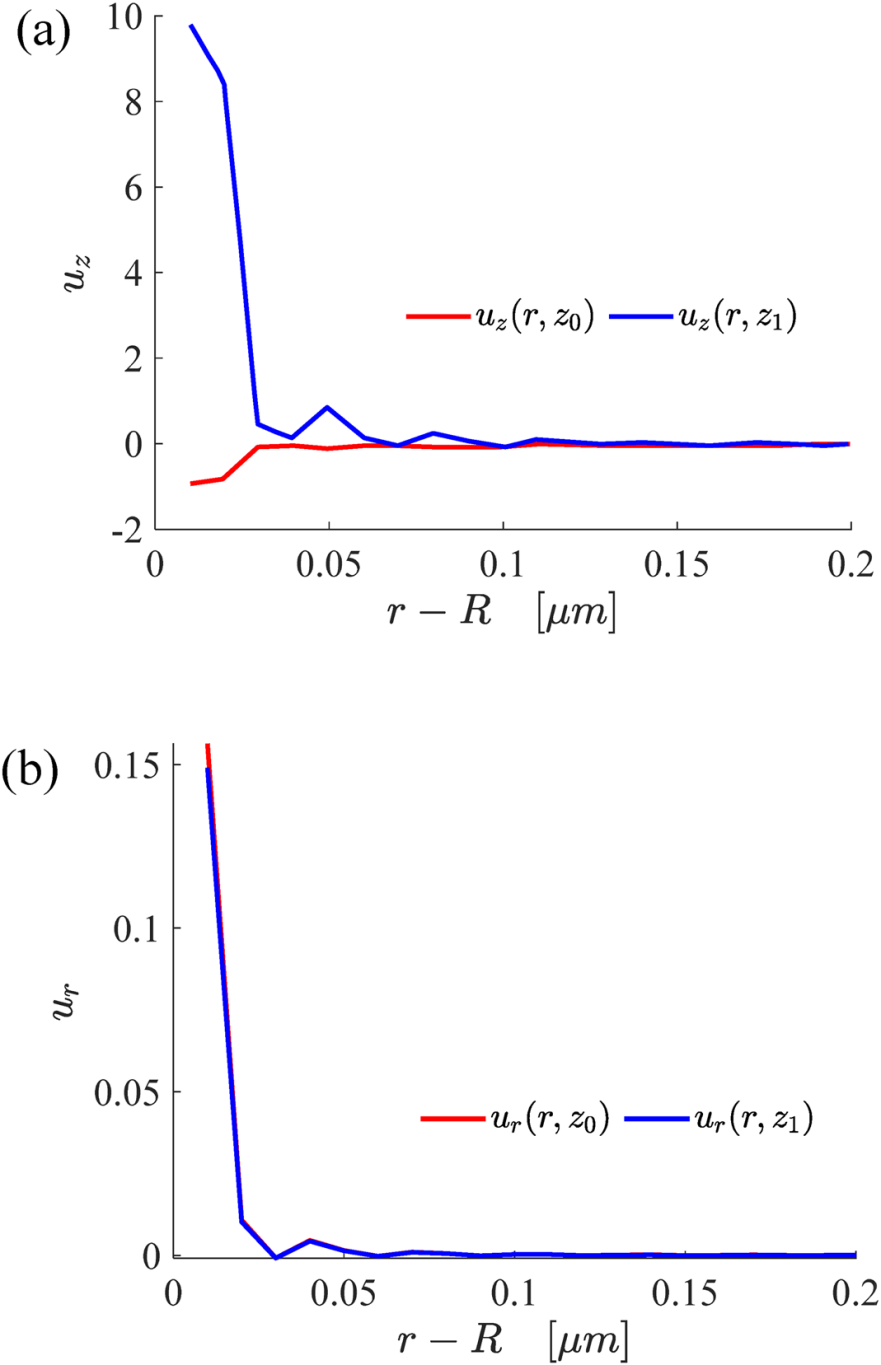
The variations of the normal (**a**) and radial (**b**) displacements of surface and inter-layer deformation.

Figure 6 shows the variations of the normal and radial displacement of the surface and inter-layer surface of the double-layer structure. It can be seen that the displacement of inter-layer surface is larger than that of surface. In addition, it also indicated that the closer the contact radius *r* is droplet radius *R*, the greater the difference in the displacement of the two layers will be. This is because the surface tension near the three contact lines has a greater influence on the deformation. As *r* is far away from *R*, the deformation of the substrate becomes weaker, finally, the displacements of the two layers almost coincide.

**Figure 7:**
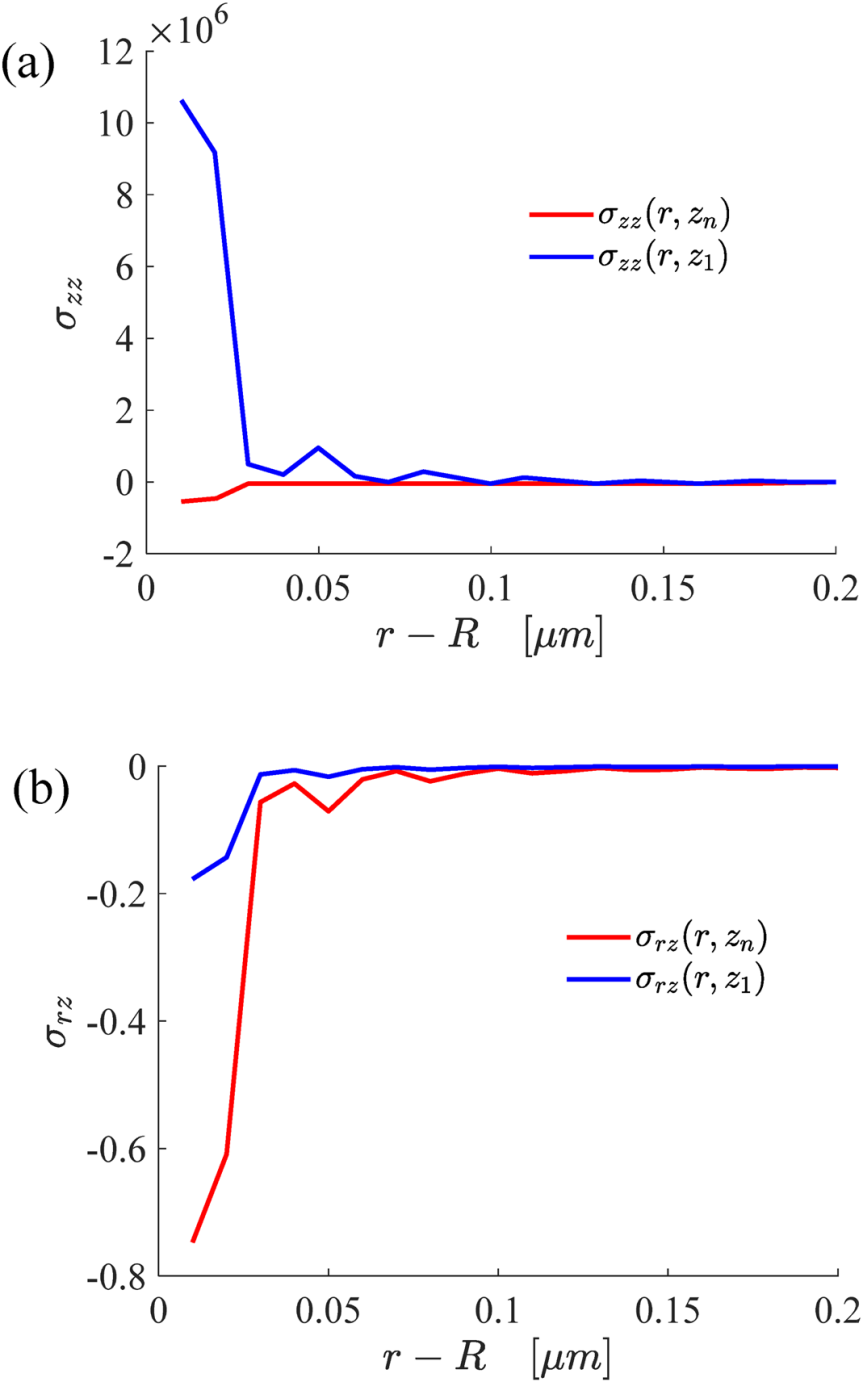
The variations of the normal stress (**a**) and radial shear stress (**b**) on the surface and interface.

Figure 7 illustrates the stress changes on the surface and inter-layer surfaces of the bilayer structure. It can be seen that the closer* r* is to *R*, the greater the difference in the stress changes between the two layers will be. On the contrary, the stress changes of the two layers almost coincide. As described in Fig. 6, the reason is that the surface tension near the three contact lines leads to the phenomenon. Figures 6 and 7 also show that the change trends of tangential displacement and tangential stress are opposite.

## Conclusion

In this paper, the wettability of a semispherical droplet on gradient layered structure was researched. Both theoretical formulas and numerical results showed that surface was producing weak radial shear stress when the droplet was placed on a thin substrate, which resulted in the very small radial deformation of the substrate. Due to unequal contact angles during the deformation process, the substrate led to tangential deformation. The numerical results also illustrated that the deformation of the substrate decreased with the increase of elastic modulus, also the variation amplitude of displacement and shear stress increased with the increase of substrate thickness, while the change of normal stress was opposite. In addition, the changes of displacement and stress between inter-layer and surface were different with the relationship of the size between contact radius and droplet radius. The contact radius was closer to droplet radius, producing the larger difference of displacement and stress between the surface and inter-layer. On the other hand, because of surface tension near the three contact lines, the changes of the two layers almost overlapped. The results of this paper have potential application value for the wetting of thin films and soft materials.

## References

[1] De Gennes, P. G. Soft Matter. *Nobel Lecture* (1991).

[2] Hamley IW (2007). Introduction to Soft Matter: Synthetic and Biological Self-Assembling Materials, Revised Edition.

[3] Bonn D, Eggers J, Indekeu J, Meunier J, Rolley E (2009). Wetting and spreading. Rev. Mod. Phys..

[4] Young T (1805). An essay on the cohesion of fluids. Philos. Trans. R. Soc. Lond..

[5] De Gennes PG, Brochard WF, Quéré D (2004). Capillarity and Wetting Phenomena: Drops, Bubbles, Pearls, Waves.

[6] Shull KR (2002). Contact mechanics and the adhesion of soft solids. Mater. Sci. Eng. R Rep..

[7] Israelachvili JN (1992). Intermolecular and Surface Forces.

[8] Wu Y, Feng J, Gao H, Feng X, Jiang L (2018). Superwettability-based interfacial chemical reactions. Adv. Mater..

[9] Zhang L, Kwok H, Li X, Yu HZ (2017). Superhydrophobic substrates from off-the-shelf laboratory filter paper: Simplified preparation, patterning, and assay application. ACS Appl. Mater. Interfaces..

[10] Xiao X, Xie W, Ye Z (2018). Preparation of corrosion-resisting superhydrophobic surface on aluminium substrate. Surf. Eng..

[11] Colburn M, Choi BJ, Sreenivasan SV, Bonnecaze RT, Grant WC (2004). Ramifications of lubrication theory on imprint lithography. Microelectron. Eng..

[12] Yang BW, Chang Q (2008). Wettability studies of filter media using capillary rise test. Sep. Purif. Technol..

[13] Hui CY, Jagota A, Lin YY, Kramer EJ (2002). Constraints on microcontact printing imposed by stamp deformation. Langmuir.

[14] Sharp KG, Blackman GS, Glassmaker NJ, Jagota A, Hui CY (2004). Effect of stamp deformation on the quality of microcontact printing: Theory and experiment. Langmuir.

[15] Bardall A, Daniels KE, Shearer M (2017). Deformation of an elastic substrate due to a resting sessile droplet. Eur. J. Appl. Math..

[16] Roman B, Bico J (2010). Elasto-capillarity: Deforming an elastic structure with a liquid droplet. J. Phys. Condens. Matter.

[17] Liu JL, Nie ZX, Jiang WG (2009). Deformation field of soft substrate induced by capillary force. Phys. B.

[18] Gurtin ME, Murdoch AI (1975). A continuum theory of elastic material surfaces. Arch. Ration. Mech. Anal..

[19] Wang GF, Feng XQ (2007). Effects of surface elasticity and residual surface tension on the natural frequency of microbeams. Appl. Phys. Lett..

[20] Style RW, Dufresne ER (2012). Static wetting on deformable substrates, from liquids to soft solids. Soft Matter.

[21] Cao Z, Dobrynin AV (2015). Polymeric droplets on soft surfaces: From Neumann’s triangle to Young’s law. Macromolecules.

[22] Jerison ER, Xu Y, Wilen LA, Dufresne ER (2011). Deformation of an elastic substrate by a three-phase contact line. Phys. Rev. Lett..

[23] Bostwick JB, Shearer M, Daniels KE (2014). Elastocapillary deformations on partially-wetting substrates: Rival contact-line models. Soft Matter.

[24] Andreotti B, Snoeijer JH (2016). Soft wetting and the Shuttleworth effect, at the crossroads between thermodynamics and mechanics. Europhys. Lett..

[25] Koursari N, Ahmed G, Tarov VM (2018). Equilibrium droplets on deformable substrates: Equilibrium conditions. Langmuir.

[26] Ye F, Di QF, Wang WC, Chen F, Chen HJ, Hua S (2018). Comparative study of two lattice Boltzmann multiphase models for simulating wetting phenomena: Implementing static contact angles based on the geometric formulation. Appl. Math. Mech. (English Ed.).

[27] Gerber J, Lendenmann T, Eghlidi H, Schutzius TM, Poulikakos D (2019). Wetting transitions in droplet drying on soft materials. Nat. Commun..

[28] Andreotti B, Snoeijer JH (2020). Statics and dynamics of soft wetting. Annu. Rev. Fluid Mech..

[29] Leong FY, Le DV (2020). Droplet dynamics on viscoelastic soft substrate: Toward coalescence control. Phys. Fluids.

[30] Dervaux J, Roche M, Limat L (2020). Nonlinear theory of wetting on deformable substrates. Soft Matter.

[31] Feng L, Li S, Li Y, Li H, Zhang L, Zhai J, Zhu D (2002). Super-hydrophobic surfaces: From natural to artificial. Adv. Mater..

[32] Wang L, Gong Q, Zhan S, Jiang L, Zheng Y (2016). Robust anti-icing performance of a flexible superhydrophobic surface. Adv. Mater..

[33] Pan E, Han F (2005). Green’s functions for transversely isotropic piezoelectric functionally graded multi-layered half spaces. Int. J. Solids Struct..

[34] Liu H, Pan E, Cai Y (2018). General surface loading over layered transversely isotropic pavements with imperfect interfaces. Adv. Eng. Softw..

[35] Cai Y, Sangghaleh A, Pan E (2015). Effect of anisotropic base/inter-layer on the mechanistic responses of layered pavements. Comput. Geotech..

